# Immobilization of muskoxen (*Ovibos moschatus*) with etorphine and xylazine

**DOI:** 10.1186/1751-0147-53-42

**Published:** 2011-06-27

**Authors:** Arnoldus Schytte Blix, Hans Lian, John Ness

**Affiliations:** 1 Department of Arctic and Marine Biology, University of Tromsø, N-9037 Tromsø, Norway

## Abstract

One hundred and thirty three "wild" muskoxen, 81 of which of known body mass, were successfully immobilized using etorphine (M99), and xylazine (Rompun^®^), delivered by use of a dart gun. A dose of 0.05 mg/kg M99, supplemented by 0.15 mg/kg Rompun was found to be very effective. This dose is much higher than currently recommended *e.g. *by Handbook of Wildlife Chemical Immobilization.

## Findings

Muskoxen (*Ovibos moschatus*) are widespread throughout the Arctic, and although there are reports of extensive use of etorphine and xylazine for the immobilization of muskoxen both in the field [1,2] and in captivity [3] systematic studies of the effects of these commonly used chemicals on these animals are few and far between. This note is based on 81 cases, out of a total of 133 successful immobilizations of altogether 34 different muskoxen of both sexes and all ages, in which age and also body mass of the animal was known by weighing subsequent to the immobilization. We arrived at doses that are much higher than those hitherto recommended [4] and we believe that our results will benefit muskoxen managers and researchers at large.

The animals belonged to the University of Tromsø and were roaming free on Rya island (69°40'N; 18°58'E) outside Tromsø, Norway, and were behaviourally wild [5].

A variable number of animals (usually 5-10) were driven into a 2 da. enclosure prior to immobilization where after the animals were approached on foot, one at a time, and subsequently a mixture of etorphine (M99; 9.8 mg/ml, Vericore Ltd., Kingsway West, UK), and xylazine (Rompun^® ^Vet; 20 mg/ml, Bayer, Leverkusen, Germany), was delivered, usually to the neck region, from a range of 20-30 m by dart syringe injection. The darts (3 ml with 1.5 × 38 mm collared needles, Dan-Inject^®^, Børkop, Denmark), were delivered by use of a CO_2_-powered Dan-Inject^®^, Børkop, Denmark, dart gun with the assistance of a Yardage Pro 600 Compact Laser Rangefinder 200600, Bushnell^®^, Cody Overland Park, Kansas, USA. Diprenorphine (12 mg/ml), Vericore Ltd., Kingsway West, UK, and atipamezole (Antisedan^®^; 5 mg/ml, Orion Pharma, Espoo, Finland), respectively, were used as antagonists, in doses relative to the anaesthetic, as recommended by the manufacturer.

Most of the immobilizations were performed during summer (May-September) over the period from 2001 to 2010, at ambient temperatures from 5°-15°C, as part of an annual wool collection, hoof care and parasite treatment program, while some were performed during winter (November-March) at subzero temperatures. The immobilized animals always lay on their side and were weighed by use of a Salter^® ^690-300S, or a Teo 500 scale, Landgraff & Flintab Vekter AS, Skedsmokorset, Norway, depending on the size of the animal. We measured rectal temperature with a digital Fluke^® ^54 II thermometer (Everett, WA, USA) and recorded heart rate and arterial oxygen saturation (SpO_2_) by use of a Rad-5v Pulse Oximeter, Masimo^® ^SET, Irvine, CA, USA, with a LNOP^® ^DCSC sensor applied to the tongue of the animal, and respiratory frequency by observation of chest movements. The animals were always darted one at a time and we never darted the next in line until the former had properly recovered. This procedure seemed to reduce the stress level to a minimum, and the rest of the herd always remained calm while an animal was under treatment. Data are given as averages ± standard deviation. "N" denotes number of different animals and "n" number of immobilizations.

The immobilizations were performed under permit from the National Animal Research Authority of Norway.

**Figure 1:**
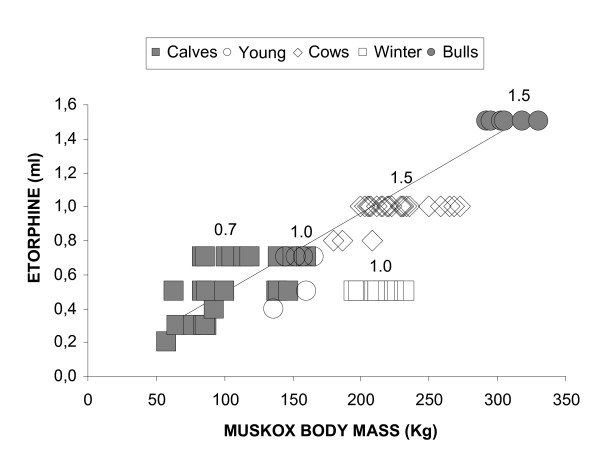
**Effective i.m. doses of etorphine (M99; ml) of a stock solution of 9.8 mg/ml for immobilization of muskoxen in relation to body mass**. Filled squares: Calves, both sexes, 1-2 years; Open circles: Animals of both sexes aged 2-3 years; Open diamonds: Mature cows in summer; Open squares: Mature cows in winter; Filled circles: Mature bulls in summer. The doses are variable, particularly in young animals, since weight was only obtained *after *the animal had been immobilized, and the variation reflects the difficulty of judging the body mass of muskoxen. The line indicates the recommended dose of 0.05 mg/kg M99. Numbers above symbols denote supplementary doses of Rompun^® ^(20 mg/ml) in ml.

In summer a dose of 0.05 mg/kg of M99 supplemented by 0.15 mg/kg of Rompun^® ^was found to be very effective (Figure 1), and suggestions for rule of thumb mixtures are given in Table 1. However, we noticed with interest that animals of all ages tolerate less during winter, when they supposedly are at their heaviest [6]. Then a 30-50% reduction of the M99 dose, supplemented with 1.0 ml (20 mg) Rompun^® ^(all ages), was indicated (Figure 1).

**Table 1:** Rule of thumb mixtures of M99 (9.8 mg/ml) and Rompun^® ^(20 mg/ml) for immobilization of muskoxen

Animals	M99 (ml)	Rompun^® ^(ml)
Mature bulls (280-320 kg)	1.5	1.5

Mature cows (190-230 kg)	1.0	1.5

2-3 years old (130-170 kg)	0.7	1.0

Calves 1-2 years (60-120 kg)	0.3-0.6	0.5-0.8

Calves < 1 year have not been chemically immobilized by us, and are instead handled with out use of drugs.

Using this recipe, the animals went down in 4 ± 2 min (time from darting to permanent recumbency), range 1-10 min (n = 81). Heart rate was 75 ± 13 beats/min (N = 8; n = 15) and oxygen saturation was as low as 58 ± 17% (N = 8; n = 15) 10 min after the drugs had been delivered. Moreover, rectal temperature was as high as 39.4 ± 0.5°C (range 38.8°C-39.9°C; N = 4; n = 5) 10-20 min after delivery of the drugs. Muscle relaxation was good, and clinical side effects were not detected, except in a single case. The animals were standing 6 ± 2 min, range 3-12 min (n = 64), after receiving antagonists into the muscles of the thigh, after being immobilized for 37 ± 10 min (n = 60), range 22-70 min.

Out of a total of 133 immobilizations only one involved complications because of an error in judging body mass of a (very lean) adult cow, which was overdosed. This resulted in respiratory arrest which was treated with the antagonists and 100 mg i.v. doxapram hydrochloride (Dopram^®^, 20 mg/ml, Wyeth Ltd., Havant, UK), which in turn resulted in hyperventilation. The animal subsequently showed signs of disorientation, but has since recovered and reproduced repeatedly. In spite of this incident, it appears that when effective dose is reached in muskoxen, the tolerance is rather high (Figure 1).

Handbook of Wildlife Chemical Immobilization [4] cites a great number of reports on the immobilization of muskoxen, of which two [1,3] seem to be relevant, of which the former seems to be the basis for its recommended dose of 0.0125 (!) mg/kg M99 and 0.1 mg/kg xylazine. Thus, Clausen et al. [1] used 0.01 mg/kg M99 and 0.1 mg/kg xylazine on wild animals, which is only 20% of the dose of M99 recommended by us, while the M99 dose used by Jingfors and Gunn [2] was about 60% of ours. The data from Clausen et al. [1] are very difficult to explain, in particular so, because they worked during July, when our data suggest the need for a relatively high dose. Jingfors and Gunn [2], on the other hand, worked during early winter (October-November), which brings their doses very close to those used by us in winter. Clausen et al. [1] also added 200 IU hyaluronidase to their mixture, while Jingfors and Gunn [2] did not. Hyaluronidase is an enzyme which facilitates the absorption rate of M99, and it is possible that it may cause a short-time knock-out effect at a relatively low dose level, while it is unlikely to give a long lasting effect. The effective use of the very low dose is therefore most likely related to the fact that their animals were only ear tagged and hence experienced a minimum of handling. It is not known, but to be expected, on the other hand, that the animals immobilized by Dieterich [3] were handled in a manner similar to ours, while his dose of M99 is still less than half of that used by us. This is difficult to explain other than that his animals may have been much tamer than ours, since it is well known that stress may increase the animal's short term tolerance appreciably. This is, of course, possible, but it is hard to imagine that the animals of Clausen et al. [1] were less excited than ours, since the former were completely wild and rounded up by use of Greenland husky dogs. Thus, since our animals would not even have shown signs of effects by the doses reported by Clausen et al. [1], the possibility remains that the M99 produced in Denmark back in the early 1980ies for reasons unknown was more potent than the drug used today.

The fact that our recommended dose of 0.05 mg/kg M99 gave excellent sedation that allowed extensive handling for an extended period and that all but one of our animals recovered from the immobilization without any sign of ill effects whatsoever does not imply that the treatment is without stress to the animal. One obviously negative effect is that respiration is initially depressed, even with the doses used by Clausen et al. [1]. This compromises both oxygenation and thermoregulation, and although it is impossible to measure rectal temperature, heart rate and arterial oxygen saturation from the very moment the animal is lying down it is quite clear from our measurements that the animals initially were both hypoxic and hyperthermic. This problem is, however, mitigated after some 10-15 min when respiration becomes normalized. We are unaware of any measurement of normal (resting) rectal temperature in adult muskoxen, but, assuming that it is similar to the 38.2°C in reindeer (*Rangifer tarandus*) [7], the body temperatures recorded in our animals were obviously suboptimal. But, it is well documented that reindeer tolerate rectal temperatures of 40.0°C for extended periods [8,9], and Clausen et al. [1] report temperatures (presumably rectal temperature) of 38.5 to 40.0°C in their muskoxen without any ill effects. In any case, this implies that immobilization of these well insulated high-arctic animals should be avoided on warm and sunny days and that the procedure should be terminated as soon as possible.

Our finding of varying tolerance to the drug throughout the year is intriguing. Nilssen et al. [10] have shown that metabolic rate is reduced in arctic ungulates due to reduced food intake in winter, and that muskoxen is one of very few species that is able to further down-regulate their metabolic rate in winter [11]. It is quite conceivable that the reduced metabolic rate may extend the effect of the drugs, but we find it unlikely that it would increase the sensitivity of the animals. Thus, while a seasonal variation in drug receptor density is possible, the seasonal variation in drug tolerance may be related to seasonal changes in total body water, as shown both in reindeer [12] and muskoxen [13].

## Conclusions

This study has shown that a dose of 0.05 mg/kg of M99 supplemented by 0.15 mg/kg of Rompun^® ^provide very effective immobilization of muskoxen during summer, while the dose of M99 should be reduced by 30-50% during winter (Figure 1).

## Competing interests

The authors declare that they have no competing interests.

## Authors' contributions

ASB designed the study, reviewed the literature and wrote the manuscript, while HL and JN assisted in the field work. All authors read and approved the final manuscript.
